# Enrichment of lithium from salt lake brine by forward osmosis

**DOI:** 10.1098/rsos.180965

**Published:** 2018-10-03

**Authors:** Jinli Li, Min Wang, Youjing Zhao, Hongjun Yang, Yuan Zhong

**Affiliations:** 1 Key Laboratory of Comprehensive and Highly Efficient Utilization of Salt Lake Resources, Qinghai Institute of Salt Lakes, Chinese Academy of Sciences, Xining 810008, People's Republic of China; 2 Key Laboratory of Salt Lake Resources Chemistry of Qinghai Province, Xining 810008, People's Republic of China

**Keywords:** forward osmosis, enrichment, salt lake brine, lithium

## Abstract

Forward osmosis (FO) is a concentration process based on the natural phenomena of osmosis. It is considered a breakthrough technology that can be potentially used for concentrating solutions and suspensions. The diluted nature of brine restricts the treatment technologies that can be applied. Then, brine concentration by FO could represent a new emerging technology enabling the application of a wider range of treatment alternatives. The performance of concentrated brine depending upon FO membranes was studied at normal temperature and pressure in this research. Cellulose triacetates on radio-frequency-weldable non-woven support (CTA-NW) and a thin-film composite with embedded polyester screen support (TFC-ES) were compared; and their orientations were considered. The brine was from Chaerhan Salt Lake after extracting potassium as the feed solution, NaCl solution or MgCl_2_ solution as the draw solution. The results indicated that CTA-NW exhibited better concentration performance than TFC-ES, while the water fluxes of the two membranes were exactly the opposite. In the case of CTA-NW in active layer facing feed solution orientation with MgCl_2_ as the draw solution, the concentration factor of Li^+^ was nearly 3.0. Quantitative structure–activity relationship of FO membranes and concentration characteristics was correlated, based on results of SEM, FTIR and contact angles studies. The concentration performance could be mainly attributed to the porosity and the thickness of FO membranes; while the water flux was dependent on the hydrophily of FO membrane surface.

## 1. Introduction

Lithium is an important rare metal, which is known as the energy metal and is promoted worldwide [1]. Lithium and lithium compounds are widely used in many industries, because they can be used as the best materials in lithium batteries [2], and important metals of the new energy and new resource [3]. Lithium is sourced mainly through pegmatite and salt lake brine [4]. Qinghai salt lakes are rich in lithium resources. To explore Li from brine has a great significance for the development of Li production industry and sustainable development [5]. Owing to low percentages of Li in Qinghai salt lakes, it is not sufficient to enrich the Li by natural evaporation. Traditionally, the brine required further concentration by evaporator after natural evaporation [6,7]. The evaporator works on electric power that is generated by the consumption of coke or natural gas, which involves not only high costs but also pollution of the environment. Therefore, it is very important to research a type of concentrator with low energy consumption, low cost and free from pollution. In recent years, many kinds of membrane technologies such as microfiltration (MF), ultrafiltration, nanofiltration (NF) and reverse osmosis (RO) have been used to treat high salinity leachate wastewater [8,9]. Studies have shown that RO and NF were capable of concentrating seawater and brines [10]. Moreover, extraction and adsorption can also be introduced to take up lithium from brine. However, RO and NF processes require external energy expenditure to force water to pass through the membranes, extraction introduces an organic reagent to cause pollution easily and adsorption has too small treatment capacity.

Forward osmosis (FO) is a newly developed membrane separation technique. Compared with the traditional pressure-driven membrane, its driving force comes from the naturally existing osmotic pressure difference between the feed solution and the draw solution [11,12]. Owing to its inherent advantages, such as low energy expenditure, low membrane fouling, simple configuration and equipment and so on [13–15], FO has been applied in various fields such as seawater desalinization [16–18], food concentration [19,20], sea water power generation [21,22] and drug delivery system [23]. However, it is still a gap for FO application in salt lake brine. Thus, FO is introduced into the system of salt lake brine because it is not only cost-effective but also pollution-free. Zhou demonstrated the crystallization of lithium carbonate (Li_2_CO_3_) microcrystals from brines of salt lakes using a FO process [24]. They found the potential and high efficiency of the FO process for crystallization of Li_2_CO_3_ from brines of salt lakes.

In this article, FO was introduced in concentrated saline lake brine. The performances of cellulose triacetate on radio-frequency-weldable non-woven support (CTA-NW) and thin-film composite with embedded polyester screen support (TFC-ES) were systematically measured. Major factors that could affect the performance of the membrane were studied, including thickness, porosity and hydrophily. The advantages and disadvantages of both of the two kinds of membranes are analysed and the application of FO in salt lake brine is proposed.

## 2. Material and methods

### 2.1. Feed solution and draw solution

 In this experiment, brine was used as the feed solution after extracting potassium from Chaerhan salt lake. The main ingredients are listed in table 1. The draw solution was NaCl solution or MgCl_2_ solution, taken from Chaerhan Salt Lake precipitates at different stages of natural evaporation.

**Table 1.** Concentrations of main ions in brine.

ion	Li^+^	Mg^2+^	K^+^	Na^+^	Cl^−^	SO_4_^2−^
concentration (mg l^−1^)	780	870	80	20	6230	30

### 2.2. Forward osmosis membrane

Preliminary tests were performed with two FO membranes: CTA-NW and TFC-ES. Both membranes were supplied by Hydration Technology Innovations, HTI (Albany, Oregon, USA).

### 2.3. Salt lake brine concentration

**Figure 1.**
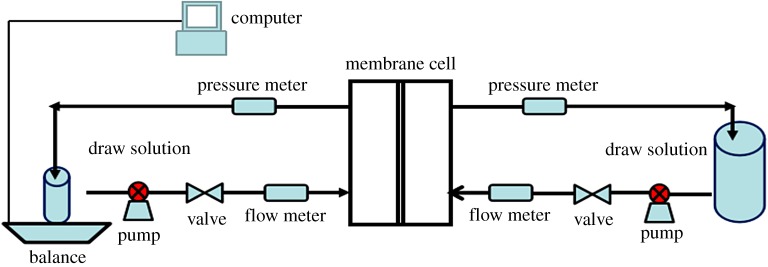
Schematic diagram of FO system for concentrating brine.

The schematic diagram of FO system in this study is shown in figure 1. The membranes were mounted in a custom-made cross-flow membrane cell with an effective membrane area of 190 cm^2^. Batch concentration assays of brine were performed, starting from samples of 1000 ml. To evaluate the two membranes, 1 mol l^−1^NaCl or 1 mol l^−1^MgCl_2_ solution was used as the draw solution. In order to keep the draw solution concentration constant, a tank containing 40 l of the draw solution was used. Such a high volume prevented changes in the draw solution exceeding 5%, despite the dilution provided by water flux from feed. The draw solution and the feed solution were continuously circulated between their containers and the respective chambers by means of peristaltic pumps at a flow of 3 l h^−1^. A computer was set to record concentration experiments. The membrane orientations were also taken into account. Namely, the active layer facing feed solution (AL-FS) and active layer facing draw solution (AL-DS) were operated at the same conditions as described above. The tests were carried out at room temperature (24–26°C).

The water flux (*J*_w_) was calculated as follows:

$$J_{w}=\frac{\Delta V}{\Delta t\cdot A_{m}},$$

where *J*_w_ is the water flux, Δ*V* is the permeated volume, Δ*t* is the time and *A*_*m*_ is the effective area of the FO membrane.

A concentration factor was calculated, as follows, to determine the concentration extent of the salt lake brine:

$$f_{c}=\frac{c_{t}}{c_{0}},$$

where *f*_c_ is the concentration factor, *c_t_* is the end concentration of ions and *c*_0_ is the initial concentration of ions.

### 2.4. Salt lake brine analysis

The concentration of salt lake brine by FO membrane was seldom reported. The present study aimed to investigate the feasibility of FO for concentrating the salt lake brine from the viewpoint of green environmental protection. Hence, salt lake brine analysis was concentrated on concentration factors of Li^+^ and Mg^2+^ in the feed solution before and after the FO filtration. The concentration of Li was analysed by an inductively coupled plasma atomic emission spectrophotometry (ICP-AES, Thermo Scientific iCAP 6500 Duo). But, the concentration of Mg^2+^ was analysed by EDTA complex formation titration.

### 2.5. Membrane characterizations

Morphological characteristics of top, bottom surfaces and cross-sectional structure of the membranes were visually examined by a scanning electron microscope (SEM, JSM-5610LV/INCA, 2.0 kV). Contact angles were measured using the captive bubble method with a computer goniometer (JCY-4). The contact angle was recorded and calculated by the software immediately once the water drop touched the membrane surface. All contact angle experiments were conducted in triplicate to confirm repeatability of the obtained data. Fourier transform infrared (FTIR) spectroscopy (Nicolet NEXUS, Thermo Electron Corporation) was used to identify organic moieties and surface chemistry.

## 3. Result and discussion

### 3.1. Membrane performance

**Figure 2.**
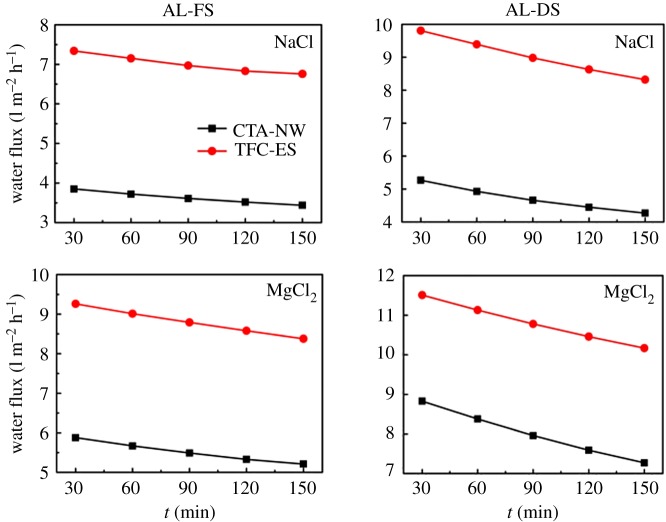
Water flux as a function time (membrane orientation: AL-FS and AL-DS).

During the process of concentrating the salt lake brine, the water flux for the two membranes was monitored using a different draw solution in both AL-DS and AL-FS orientation. As shown in figure 2, the initial membrane flux of AL-DS is higher than that of AL-FS, which was consistent with other FO membrane reports [25,26]. It can be explained by the difference of dilutive concentration polarization of the draw solution between two membrane orientations. Figure 2 also shows that with increasing FO concentration time, the membrane flux gradually declined in both AL-DS and AL-FS orientations at different draw solutions, which is mainly attributed to a decrease in the overall driving force due to either the increasing salinity in the feed solution or the dilution in the draw solution. Moreover, it is clear that TFC-ES presents a higher water flux when compared with CTA-NW in both AL-DS and AL-FS orientations, especially MgCl_2_ as the draw solution. These results are in agreement with reports in the literature which indicates that TFC membrane would provide higher flux than CTA membrane [27]. Based on these results, with regard to water flux, TFC-ES in AL-DS orientation at MgCl_2_ draw solution works better.

The research was yet to take the concentration factors of Li^+^ and Mg^2+^ into account over and above water flux. Concentration factors represent the times the value of a certain parameter increases, as a result of the concentration process. Table 2 presents the concentration of Li^+^ and Mg^2+^ in the feed solution before and after the FO process. The assays started with a concentration of 0.78 g l^−1^ and 0.87 g l^−1^ for Li^+^ and Mg^2+^, respectively, with the ratio of molar mass around 3.05 : 1.0. After the operation, the ratio between those two ions became 2.4–2.8: 1.0, suggesting that Li^+^ penetrated easier than Mg^2+^. In theory, FO membrane does not selectively transport ions and it can reject a wide range of ions. Both Li^+^ and Mg^2+^ should stay in the feed solution and the ratio remain unchanged. This change and imbalance were probably due to the FO membrane, which more retarded with Mg^2+^ passing through. The smaller the diameter of ion, the bigger the penetration efficiency. In addition, lesser decay of flux of membrane illustrated that CTA-NW membrane had a better anti-pollution effect. The nature brine contains some organics, such as humic acids, which could well lead to the fouling of the membrane. With regard to concentration factors, CTA-NW in AL-FS orientation works better.

**Table 2.** Concentration of feed solution before and after the FO process.

			concentration			
			before		after	
sample	membrane orientation	draw solution	Li^+^(g l^−1^)	Mg^2+^(g l^−1^)	Li^+^(g l^−1^)	Mg^2+^(g l^−1^)
CTA-NW	AL-FS	NaCl	0.78	0.87	1.41	1.92
		MgCl_2_	0.78	0.87	1.83	2.44
	AL-DS	NaCl	0.78	0.87	1.32	1.63
		MgCl_2_	0.78	0.87	1.67	2.01
TFC-ES	AL-FS	NaCl	0.78	0.87	1.26	1.85
		MgCl_2_	0.78	0.87	1.52	1.97
	AL-DS	NaCl	0.78	0.87	1.20	1.45
		MgCl_2_	0.78	0.87	1.41	1.75

FO is a concentration process based on the natural phenomenon of osmosis. In the absence of any external pressure, FO uses naturally generated driving force between the draw solution and the feed solution. Choosing a reasonable draw solution is even more important. These displays showed that the water flux is greater when MgCl_2_ was used as the draw solution compared with NaCl in all cases. The reason is that MgCl_2_ has higher osmotic pressure, contributing to the larger driving force. Mg^2+^, on the other hand, as a divalent cation with larger radius gave a negative penetrability [28].

Above all, based on concentration factors of Li^+^ and Mg^2+^ in the feed solution before and after the FO filtration, CTA-NW in AL-FS orientation at MgCl_2_ is more adaptable to a further study of salt lake brine concentrating rather than the other one.

### 3.2. Membrane microscopic observation

FO membrane has an asymmetric structure composed of a dense active layer and a porous support layer. Generally, the active layer determines the membrane selectivity and also greatly affects the membrane permeability and anti-pollution features. The support layer affects not only water migration, but also separation properties. Arguably, the properties of FO membrane mainly depended on the eigen structure of the support layer, such as thickness, porosity and hydrophilicity or hydrophobicity [29,30].

**Figure 3.**
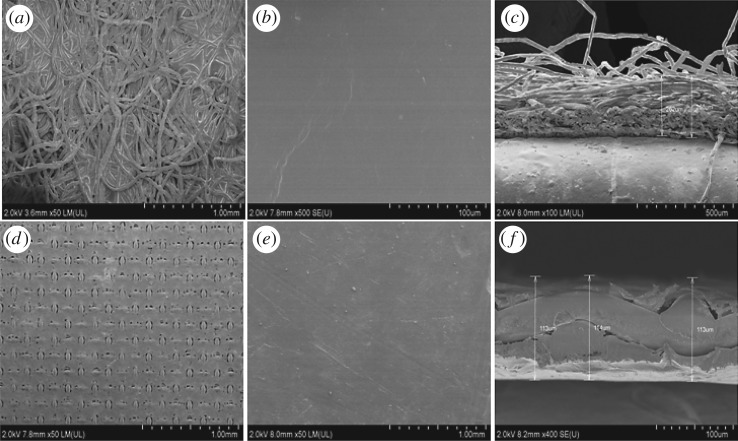
SEM: (*a*) support layer of CTA-NW; (*b*) active layer of CTA-NW; (*c*) cross-section of CTA-NW; (*d*) support layer of TFC-ES; (*e*) active layer of TFC-ES and (*f*) cross-section of TFC-ES.

Figure 3 shows that CTA-NW membrane is formed by two layers. Figure 3*a* is a supported layer with polyester non-woven fabric structure, which consists of polyethylene-coated polyester fibres. Figure 3*b* is the side of cellulose acetate that was smooth tight. In the cross-section of the membrane, figure 3*c*, the thickness of the membrane was 80–120 µm, the layer of cellulose acetate was above 80 µm. TFC-ES membrane has rather complicated structures; the polyester screen was sandwiched in between two films. Figure 3*f* shows the cross-section of the membrane; the thickness of the membrane was 110–120 µm (figure 3*d*). The functional layer was smooth, while the support layer was rough. Figure 3*d* exhibits the TFC-ES membrane functional layer with many pores, which have been caused by cracking during the drying process. By contrast, the functional layer of CTA-NW was more tight and durable and the support layer with low porosity, so the desalination efficiency of CTA-NW was high. Overall, CTA-NW was more suitable to concentrate Li^+^ and Mg^2+^ from brine.

### 3.3. Contact angle measurement

The surface property of a membrane is an important research; it can impact the performance of operation and physics. Hydrophilicity and hydrophobicity of a membrane can by deduced by contact angle measurement; the smaller the angle, the more hydrophilic. The membrane with strong hydrophilicity is difficult to react with any other material, which made the pollution difficult to deposit and prolong the service life. The FO membrane is asymmetric; there are different hydrophilicities between the functional layer and the support layer.

**Figure 4.**
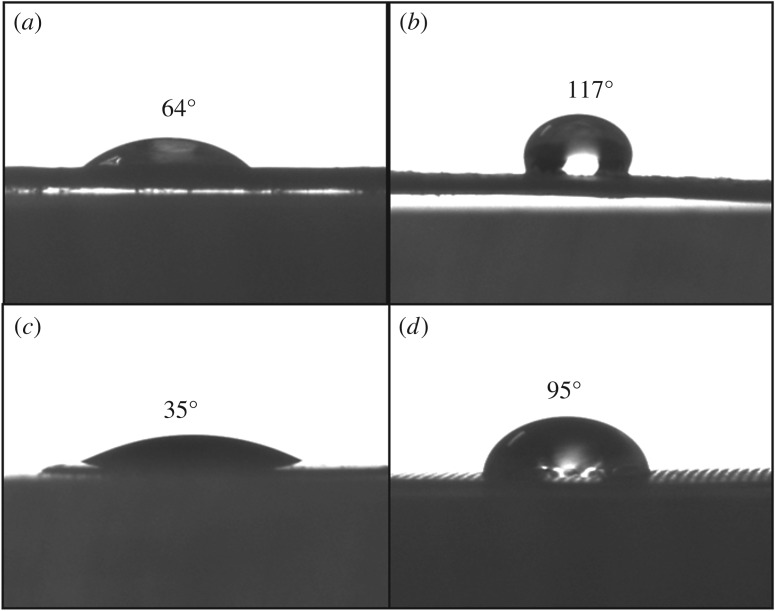
Contact angle: (*a*) active layer of CTA-NW; (*b*) support layer of CTA-NW; (*c*) active layer of TFC-ES and (*d*) support layer of TFC-ES.

Figure 4 shows the contact angle measurements of both the active layer and support layer of two membranes. For either CTA-NW or TFC-ES, the contact angle of active layer is smaller than that of support layer, suggesting that the active layer had better hydrophilicity, so that the AL-DS orientation made the water flux higher. The results of the contact angle studies are consistent with membrane fluxes. Previous studies indicated that the hydrophilicity of the support layer had a great effect upon membrane flux; the stronger the hydrophilicity, the higher the water flux [29,31]. However, compared with the ingredients of the draw solution, those of the feed solution are more complex and easily contribute to pollution of the support layer. So, it is more reasonable to make the functional layer toward a material solution, namely AL-FS orientation.

### 3.4. Infrared feature surface chemistry of forward osmosis membrane

**Figure 5.**
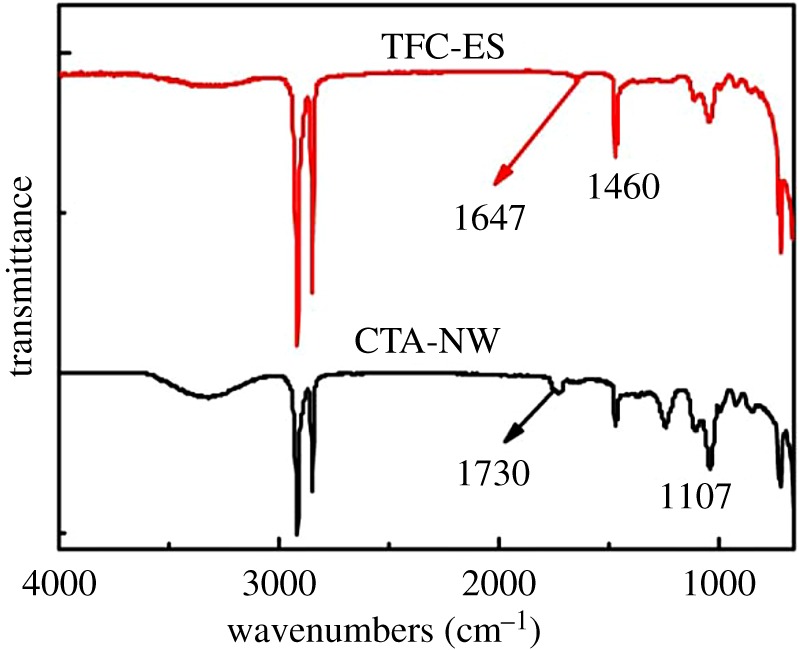
Infrared (IR) spectra of CTA-NW and TFC-ES.

FTIR spectroscopy allowed us to investigate the membrane surface structure. A comparison of FTIR spectra between CTA-NW and TFC-ES was used to determine the organic composition, as shown in figure 5. Both of them display the same peaks at around 2980 cm^−1^ represented CH_3_. However, when it comes down to 1000–1800 cm^−1^, they have different characteristic peaks. For CTA-NW, the strong absorptions at 1107 and 1730 cm^−1^ are characteristic peaks, which are, respectively, characteristic band of C = O and C-O-C stretching vibrations in an aromatic polymer. For TFC-ES, the peaks at 1647 and 1460 cm^−1^ represented the amide and substituted benzene of polyurethane, respectively. In terms of the functional group, acylamino in TFC-ES had stronger hydrophilic than ester in CTA-NW, which was substantiated with higher water flux. However, the TFC-ES membrane belonged to a kind of polyester (of polyamide) composite membrane. It has inherent disadvantages such as weak anti-pollution capacity and poor chlorine resistance that cannot resist oxidization and depositing [32,33]. The brine contains a larger quantity of Cl^−^, so CTA-NW is more appropriate.

## 4. Conclusion

In this study, we integrated FO process into the concentration of salt lake brine. The results showed that Li^+^ and Mg^2+^ could be effectively concentrated by FO and gradually accumulated in the feed solution. The concentration factors of Li^+^ and Mg^2+^ reached 2.3 and 2.8, respectively, in AL-FS orientation with MgCl_2_ as the draw solution. A gradual decrease in water flux was observed in every case, which is attributed to the drop of the overall driving force caused by the build-up of salinity in the feed solution and the dilution in the draw solution. AL-FS outperformed AL-DS in terms of concentration factors, while in terms of water fluxes it was exactly opposite. But now the core technology of FO cannot yet satisfy the targeted goals, but its use cannot be hindered in exploiting the brine. Generally, with the intensive study on FO, it will widely be used in exploiting the salt lake brine.

## Data accessibility

Our data are deposited at Dryad Digital Repository: http://dx.doi.org/10.5061/dryad.r1m29fm [34].

## References

[1] An JW, Kang DJ, Tran KT, Kim MJ, Lim T, Tran T 2012 Recovery of lithium from Uyuni salar brine. Hydrometallurgy 117, 64–67. (10.1016/j.hydromet.2012.02.008)

[2] Ilic D, Kilb M, Holl K, Praas HW, Pytlik E 1999 Recent progress in rechargeable nickel/metal hydride and lithium-ion miniature rechargeable batteries. J. Power Sources 80, 112–115. (10.1016/S0378-7753(99)00067-1)

[3] Rahman MM, Wang JZ, Zeng R, Wexler D, Liu HK 2012 LiFePO_4_-Fe_2_P-C composite cathode: an environmentally friendly promising electrode material for lithium-ion battery. J. Power Sources 206, 259–266. (10.1016/j.jpowsour.2012.01.119)

[4] Wang XL, Li JL, Zhang MJ 2001 Energetic metal of the 21th century: the use of metal lithium in nuclear fusion. Gold. J. 3, 249–252. (doi:1008-8067(2001)04-0249-04)

[5] Ma PH 2009 Sustainable exploitation and comprehensive utilization of salt lake resources in China. Prog. Chem. 21, 2349–2357.

[6] Liu YH, Deng TL 2006 Progresses on the process and technique of lithium recovery from salt lake brines around the world. World Sci.-Tech. R&D 28, 69–75.

[7] Wang WD, Cao Q 2010 Production process and current situation of lithium carbonate extraction from salt lake brine in China. J. Salt Lake Res. 18, 52–56. (doi:1008-858X(2010)04-0052-10)

[8] Rukapan W, Khananthai B, Srisukphun T, Chiemchaisri W, Chimchaisri C 2015 Comparison of reverse osmosis membrane fouling characteristics in full-scale leachate treatment systems with chemical coagulation and microfiltration pre-treatments. Water Sci. Technol. 71, 580–587. (10.2166/wst.2014.468) 25746651

[9] Kuusik A, Pachel K, Kuusik A, Loigu E, Tang WZ 2014 Reverse osmosis and nanofiltration of biologically treated leachate. Environ. Technol. 35, 2416–2426. (10.1080/09593330.2014.908241) 25145196

[10] Somrani A, Hamzaoui AH, Pontie M 2013 Study on lithium separation from salt lake brines by nanofiltration (NF) and low pressure reverse osmosis (LPRO). Desalination 317, 184–192. (10.1016/j.desal.2013.03.009)

[11] Cath TY, Childress AE, Elimelech M 2006 Forward osmosis: principles, applications, and recent developments. J. Membr. Sci. 281, 70–87. (10.1016/j.memsci.2006.05.048)

[12] Babu BR, Rastogi NK, Raghavarao KSMS 2006 Effect of process parameters on transmembrane flux during direct osmosis. J. Membr. Sci. 280, 185–194. (10.1016/j.memsci.2006.01.018)

[13] Cath TY, Gormly S, Beaudry EG, Flynn MT, Adams VD, Childress AE 2005 Membrane contactor processes for wastewater reclamation in space. Part I. Direct osmotic concentration as pretreatment for reverse osmosis. J. Membr. Sci. 257, 85–98. (10.1016/j.memsci.2004.08.039)

[14] Melo M, Schluter H, Ferreira J, Magda R, Junior A, de Aquino O 2010 Advanced performance evaluation of a reverse osmosis treatment for oilfield produced water aiming reuse. Desalination 250, 1016–1018. (10.1016/j.desal.2009.09.095)

[15] McGinnis RL, Elimelech M 2008 Global challenges in energy and water supply: the promise of engineered osmosis. Environ. Sci. Technol. 42, 8625–8629. (10.1021/es800812m) 19192773

[16] Patel-Predd P 2006 Water desalination takes a step forward. Environ. Sci. Technol. 40, 3454–3455. (10.1021/es063016j) 16786679

[17] Kravath RE, Davis JA 1975 Desalination of seawater by direct osmosis. Desalination 16, 151–155. (10.1016/S0011-9164(00)82089-5)

[18] Kessler JO, Moody CD 1976 Drinking water from sea water by forward osmosis. Desalination 18, 297–306. (10.1016/S0011-9164(00)84119-3)

[19] Petrotos KB, Lazarides HN 2001 Osmotic concentration of liquid foods. J. Food Eng. 49, 201–206. (10.1016/S0260-8774(00)00222-3)

[20] Wrolstad RE, McDanniel MR, Durst RW, Micheals N, Lampi KA, Beaudry EG 1993 Composition and sensory characterization of red raspberry juice concentrated by direct-osmosis or evaporation. J. Food Sci. 58, 633–637. (10.1111/j.1365-2621.1993.tb04344.x)

[21] Loeb S 1998 Energy production at the Dead Sea by pressure-retarded osmosis: challenge or chimera? Desalination 120, 247–262. (10.1016/S0011-9164(98)00222-7)

[22] Loeb S, Hassen FV, Shahaf D 1976 Production of energy from concentrated brines by pressure-retarded osmosis II: experimental results and projected energy costs. J. Membr. Sci. 1, 246–269. (10.1016/S0376-7388(00)82271-1)

[23] Su YC, Lin LA 2004 A water-powered micro drug delivery system. J. Microelectronic Syst. 13, 75–82. (10.1109/JMEMS.2003.823215)

[24] Zhou H 2017 Optimisation of crystallisation parameters for lithium carbonate microcrystals based on forward osmosis (FO) process. Mater. Res. Innovations 21, 1–9. (10.1179/1433075X15Y.0000000029)

[25] Lutchmiah K, Corelissen ER, Harmsen DJH, Post JW, Lampi K, Ramaekers H, Rietveld LC, Roest K 2011 Water recovery from sewage using forward osmosis. Water Sci. Technol. 64, 1443–1449. (10.2166/wst.2011.773) 22179641

[26] Linares RV, Li ZY, Abu-Ghdaib M, Wei CH, Amy G, Vrowenveder JS 2013 Water harvesting from municipal wastewater via osmotic gradient: an evaluation of process performance. J. Membr. Sci. 447, 50–56. (10.1016/j.memsci.2013.07.018)

[27] Ortega-Bravo JC, Ruiz-Filippi G, Donoso-Bravo A, Reyes-Cauiupan IE, Jeison D 2016 Forward osmosis: evaluation thin-film-composite membrane for municipal sewage concentration. Chem. Eng. J. 306, 531–537. (10.1016/j.cej.2016.07.085)

[28] Shi J, Yuan Q, Gao CJ 2001 Membrane technology handbook. Beijing, China: Chemical Industry Press. [In Chinese.]

[29] McCutcheon JR, Elimelech M 2008 Influence of membrane support layer hydrophobicity on water flux in osmotically driven membrane processes. J. Membr. Sci. 318, 458–466. (10.1016/j.memsci.2008.03.021)

[30] Loeb S, Titelman L, Korngold E, Freiman J 1997 Effect of porous support fabric on osmosis through a Loeb-Sourirajan type asymmetric membrane. J. Membr. Sci. 129, 243–249. (10.1016/S0376-7388(96)00354-7)

[31] Bui NN, McCutcheon JR 2013 Hydrophilic nanofibers as new supports for thin film composite membranes for engineered osmosis. Environ. Sci. Technol. 47, 1761–1769. (10.1021/es304215g) 23234259

[32] Glater J, Hong S, Elimelech M 1994 The search for a chlorine-resistant reverse-osmosis membrane. Desalination 95, 325–345. (10.1016/0011-9164(94)00068-9)

[33] Konagaya S, Tokai M, Kuzumoto H, Watanabe O 2000 New reverse osmosis membrane materials with higher resistance to chlorine. J. Appl. Polymer Sci. 75, 1357–1364.

[34] Li J, Wang M, Zhao Y, Yang H, Zhong Y 2018 Data from: Enrichment of lithium from salt lake brine by forward osmosis *Dryad Digital Repository*. (10.5061/dryad.r1m29fm) PMC6227972 30473842

